# Supplementary material for: Empirical methods for controlling false positives and estimating confidence in ChIP-Seq peaks
Source: BMC Bioinformatics. 2008 Dec 5;9:523. doi: 10.1186/1471-2105-9-523 (PMC2628906; doi:10.1186/1471-2105-9-523)
Supplement: Additional File 1 — A variety of html documents from the USeq web site detailing the available applications, their best usage, output file type descriptions, command line menus, etc. [file 1471-2105-9-523-S1.zip › USeqUserGuides/JavaDoc.zip!/JavaDoc/trans/qc/StatFlag.html]

StatFlag


|  |  |  |
| --- | --- | --- |
| **Overview** \| **Package** \| **Class** \| **Use** \| **Tree** \| **Deprecated** \| **Index** \| **Help** | |  |
| **PREV CLASS**   NEXT CLASS | **FRAMES**    **NO FRAMES**     **All Classes** |  |
| SUMMARY: NESTED \| FIELD \| CONSTR \| METHOD | DETAIL: FIELD \| CONSTR \| METHOD |  |


---


## trans.qc Class StatFlag

```
java.lang.Object
  trans.qc.StatFlag
```

---

``` public class StatFlag extends java.lang.Object ```

Info related to a particular statistic used to measure a cel file.

---

**Constructor Summary**

`StatFlag(java.lang.String name,
double minimum,
double maximum)`


| **Method Summary** | |
| --- | --- |
| `double` | `getMaximum()` |
| `double` | `getMedian()` |
| `double` | `getMinimum()` |
| `java.lang.String` | `getName()` |
| `double` | `getStndDev()` |
| `void` | `rangeCheck(double value, java.lang.StringBuffer sb)`             Checks min max to see if value is contained withing, adds a 'Warning' line to the StringBuffer if so. |
| `void` | `setCheckMax(boolean checkMax)` |
| `void` | `setCheckMin(boolean checkMin)` |
| `void` | `setMaximum(double maximum)` |
| `void` | `setMedian(double median)` |
| `void` | `setMinimum(double minimum)` |
| `void` | `setName(java.lang.String name)` |
| `void` | `setStndDev(double stndDev)` |
| `java.lang.String` | `toString()` |

**Methods inherited from class java.lang.Object**

`equals, getClass, hashCode, notify, notifyAll, wait, wait, wait`

**Constructor Detail**

### StatFlag

```
public StatFlag(java.lang.String name,
                double minimum,
                double maximum)
```


**Method Detail**

### rangeCheck

```
public void rangeCheck(double value,
                       java.lang.StringBuffer sb)
```

:   Checks min max to see if value is contained withing, adds a 'Warning' line to the StringBuffer if so.
    Can disable checking minimum by setting boolean.

---


### toString

```
public java.lang.String toString()
```

:   **Overrides:**: `toString` in class `java.lang.Object`
:   **Returns:**: name median stndDev min max adjustor

---


### getMaximum

```
public double getMaximum()
```

---


### setMaximum

```
public void setMaximum(double maximum)
```

---


### getMedian

```
public double getMedian()
```

---


### setMedian

```
public void setMedian(double median)
```

---


### getMinimum

```
public double getMinimum()
```

---


### setMinimum

```
public void setMinimum(double minimum)
```

---


### getName

```
public java.lang.String getName()
```

---


### setName

```
public void setName(java.lang.String name)
```

---


### setCheckMin

```
public void setCheckMin(boolean checkMin)
```

---


### setCheckMax

```
public void setCheckMax(boolean checkMax)
```

---


### getStndDev

```
public double getStndDev()
```

---


### setStndDev

```
public void setStndDev(double stndDev)
```


---


|  |  |  |
| --- | --- | --- |
| **Overview** \| **Package** \| **Class** \| **Use** \| **Tree** \| **Deprecated** \| **Index** \| **Help** | |  |
| **PREV CLASS**   NEXT CLASS | **FRAMES**    **NO FRAMES**     **All Classes** |  |
| SUMMARY: NESTED \| FIELD \| CONSTR \| METHOD | DETAIL: FIELD \| CONSTR \| METHOD |  |


---
